# Ruxolitinib significantly enhances *in vitro* apoptosis in Hodgkin lymphoma and primary mediastinal B-cell lymphoma and survival in a lymphoma xenograft murine model

**DOI:** 10.18632/oncotarget.24267

**Published:** 2018-01-18

**Authors:** Sanghoon Lee, Tishi Shah, Changhong Yin, Jessica Hochberg, Janet Ayello, Erin Morris, Carmella van de Ven, Mitchell S. Cairo

**Affiliations:** ^1^ Department of Pediatrics, New York Medical College, Valhalla, NY 10595, USA; ^2^ Department of Cell Biology and Anatomy, New York Medical College, Valhalla, NY 10595, USA; ^3^ Department of Medicine, Pathology, Microbiology and Immunology New York Medical College, Valhalla, NY 10595, USA

**Keywords:** ruxolitinib, survival, Hodgkin lymphoma, primary mediastinal B-cell lymphoma

## Abstract

Hodgkin lymphoma (HL) and primary mediastinal B-cell lymphoma (PMBL) share similar molecular features by gene expression profiling. Frequent gains of chromosome 9p exhibit higher Janus Kinase 2 (JAK2) transcript levels with increased JAK2 activity, suggesting aberrant activity of JAK2 and STAT pathways. This signaling pathway alteration may in part play an important role in the pathogenesis and/or chemoradiotherapy resistance in HL and PMBL. Ruxolitinib is a potent and selective JAK1/JAK2 inhibitor, with activity against myeloproliferative neoplasms (MPNs) including those harboring the JAK2V617F mutation. We investigated the *in vitro* and *in vivo* efficacy of ruxolitinib and changes in downstream signaling pathways in HL and PMBL. We demonstrated that ruxolitinib significantly inhibited STAT signaling in both HL and PMBL with constitutively active JAK2 signaling. We also observed that ruxolitinib significantly induced *in vitro* anti-proliferative effects (*p <* 0.05) and increased programmed cell death (*p <* 0.05) against both HL and PMBL cells. Importantly, ruxolitinib significantly inhibited tumor progression by bioluminescence (*p <* 0.05) and significantly improved survival in HL (*p =* 0.0001) and PMBL (*p <* 0.0001) xenograft NSG mice. Taken altogether, these studies suggest that ruxolitinib may be a potential adjuvant targeted agent in the therapeutic approach in patients with high risk HL and PMBL.

## INTRODUCTION

Hodgkin lymphoma (HL) and primary mediastinal large B-cell lymphoma (PMBL) are two of the most common malignancies among adolescents and young adults (AYA). HL and PMBL share similar molecular features [1, 2]. HL has a bimodal age distribution, with a first peak at 15–35 years of age and second peak after 50 years of age [3]. Although HL represents approximately 4–5% of all cancers in children younger than 15 years of age, HL is the most common cancer in the 15–35 years AYA group, with an incidence of about 16% [4]. The prognosis is excellent in AYA with HL, with 10 year overall survival rates approximately 85–95% [5]. However, patients with an early relapse (≤12 months from diagnosis) and/or have metastatic extra-nodal disease at relapse/progression have a dismal prognosis [6, 7]. Furthermore, there are significant late effects secondary to chemo-radiotherapy in patients with HL, including cardiac and pulmonary toxicity, infertility and increased risk of secondary malignancy, which affect the long-term event-free survival (EFS) especially in AYA [8, 9]. Therefore, new targeted agents are needed to avoid late effects in HL and improve the outcomes in patients with high risk of relapse/progression.

PMBL was previously considered a subtype of diffuse large B-cell lymphoma (DLBCL) and is now classified as distinct mature B-cell lymphoma [10]. PMBL is a rare malignancy that, although more common among adults in their third and fourth decades of life, is also commonly seen in AYA, with a female predominance [4, 11–13]. PMBL represents approximately 4% of mature B-cell non-Hodgkin lymphoma in patients ≤18 years of age [14–17]. Although considered a mature B-cell lymphoma, we have identified that children and adolescents with newly diagnosed PMBL have a significantly decreased EFS compared to children and adolescents with other forms of DLBCL treated with similar therapy [16]. HL and PMBL also share similar cytogenetic abnormalities, namely chromosome 9p and 2p gains [18, 19] including rearrangement of chromosome 13 [20, 21]. Gain in 9p has been demonstrated to be associated with an upregulation in the expression of the Janus Kinase (JAK) 2 (JAK2) gene in about 50% of patients, leading to phosphorylation and activation of transcription factor signal transducer and activator of transcription (STAT) 6 [2, 22, 23]. Gain in 2p16 region has been associated with duplication of REL proto-oncogene that encodes for nuclear factor kappa B (NFKB) transcription factor [22]. *PTPN1* mutations have also been shown recently in both HL and PMBL, leading to hyper-phosphorylation in JAK-STAT pathway [24]. HL and PMBL thus exhibit higher JAK2 transcript levels with increased JAK2 activity [25], suggesting aberrant activity of JAK2 and STAT pathways may in part play an important role in the pathogenesis and/or resistance in AYA HL and PMBL. Inhibition of JAK2 by fedratinib in HL and mediastinal large B-cell lymphoma (MLBL) has been significantly associated with an inhibition of cell proliferation and decreased growth in MLBL xenografted non-obese diabetic severe combined immunodeficiency gamma (NSG) mice, further establishing the importance of JAK2 activation in these tumors [26].

The role of JAK2 inhibitors like ruxolitinib and fedratinib has been studied in patients with myelofibrosis and myeloproliferative neoplasms (MPNs) that consistently exhibit dysregulation of the JAK1/JAK2 pathway [27–29]. Ruxolitinib is a potent and selective ATP-competitive inhibitor of JAK1 and JAK2 kinases against MPNs including those with a JAK2^V617F^ mutation. Ruxolitinib also inhibits JAK2/STAT5 signaling *in vitro* and in murine models of MPNs [30]. It is worthy to note that ruxolitinib is associated with marked and durable clinical benefits in patients with myelofibrosis [31]. Interestingly the clinical benefit in MPN patients was achieved irrespective of *JAK2*^*V617F*^ status, which suggests that the pathophysiological consequence of hyperactivity of the JAK/STAT pathway can be downregulated with ruxolitinib therapy [32].

We hypothesize that ruxolitinib may potentially be an effective therapeutic agent, in part by inducing targeted programmed cell death in both HL and PMBL. Therefore, we investigated the *in vitro* and *in vivo* efficacy of ruxolitinib against HL and PMBL cells in an immunodeficient mouse model (NSG) xenografted with human HL and PMBL and its *in-vitro* effects on downstream protein signaling pathways.

## RESULTS

### Effect of ruxolitinib on the JAK2/STAT signaling pathway in HL cells

**Figure 1:**
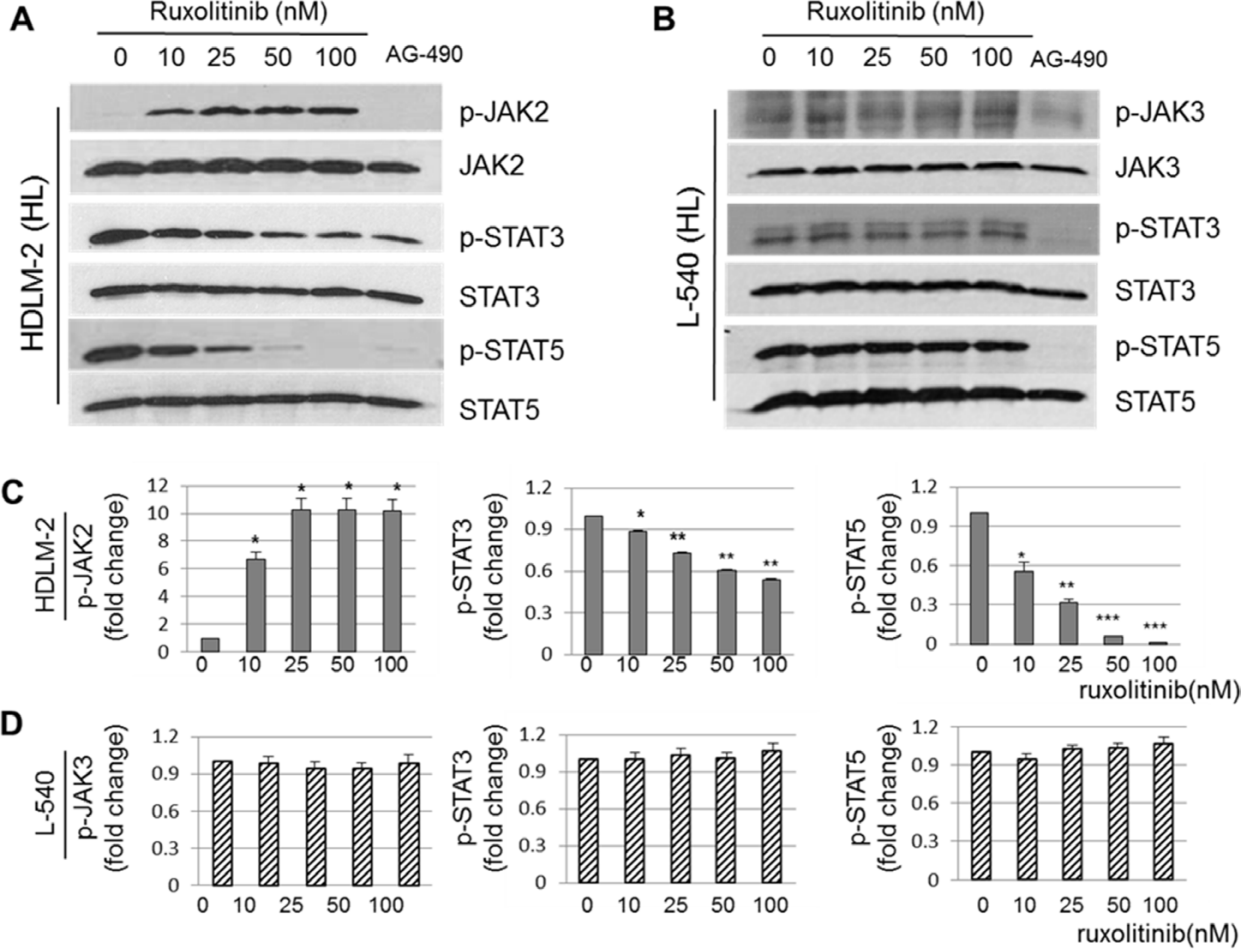
Selective activity of ruxolitinib on the JAK2/STAT signaling pathway in HL cells Ruxolitinib (0, 10, 25, 50, and 100 nM) treated HDLM-2 and L-540 HL cells were collected and the lysates were separated in SDS-PAGE gels (**A and B**, respectively). The pan-JAK inhibitor, AG490 was used as a negative control treatment in each cell. Significant differences of p-JAK2, p-STAT3 and p-STAT5 with the quantification on the intensity of the bands by western blotting in HDLM-2 **(C)** (*n =* 3) and L-540 **(D)** (*n =* 3) cells. Data are represented as the mean ± SD. ^*^p < 0.005; ^**^p < 0.0005; ^***^p < 0.0001.

The effect of ruxolitinib on the JAK2/STAT signaling pathways was examined measuring the phosphorylation status of JAK2 and its downstream substrates in HL cell lines. First, we observed that increasing concentrations of ruxolitinib (10-100 nM) for 24 h significantly inhibited downstream active phosphorylated STAT3 (p-STAT3, *p <* 0.005 at 10 nM, and *p <* 0.0005 at 25 - 100 nM) and phosphorylated STAT5 (p-STAT5, *p <* 0.005 at 10 nM, *p <* 0.0005 at 25 nM, and *p <* 0.0001 at 50 and 100 nM) in a dose-dependent manner in HDLM-2 cells (Figure 1A and 1C), whereas, total STAT3 and STAT5 levels remained unchanged at the concentrations up to 100 nM (Figure 1B and 1D). Similarly, no differences in expression of p-STAT3 and p-STAT5 were observed at different time points up to 100 nM ruxolitinib treated HDLM-2 cells at 48 and 72 hours (Data not shown). The dose escalation of ruxolitinib demonstrated an increase of the level of phosphorylated JAK2 (p-JAK2) in HDLM-2 cells. Conversely, we found no inhibitory effects of ruxolitinib at concentrations up to 100 nM in another HL cell line, L-540, which contains constitutively-active forms of JAK3, but not JAK2. In contrast, the pan-JAK inhibitor AG490 non-selectively inhibited the phosphorylation levels of these p-JAK2, p-STAT3 and p-STAT5 tested in both of HDLM-2 cells and L-540 cells. These results suggest that ruxolitinib inhibits JAK2/STAT signaling by blocking of downstream of the phosphorylation of STAT3 and STAT5 and that ruxolitinib shows selective activity of JAK2 against JAK3 in HL cell lines which is consistent with other studies suggesting that ruxolitinib is a JAK1/JAK2 inhibitor with marked selectivity over JAK3.

### Effect of ruxolitinib on phosphorylation of STAT-3, -5 and -6 in HL and PMBL cells

**Figure 2:**
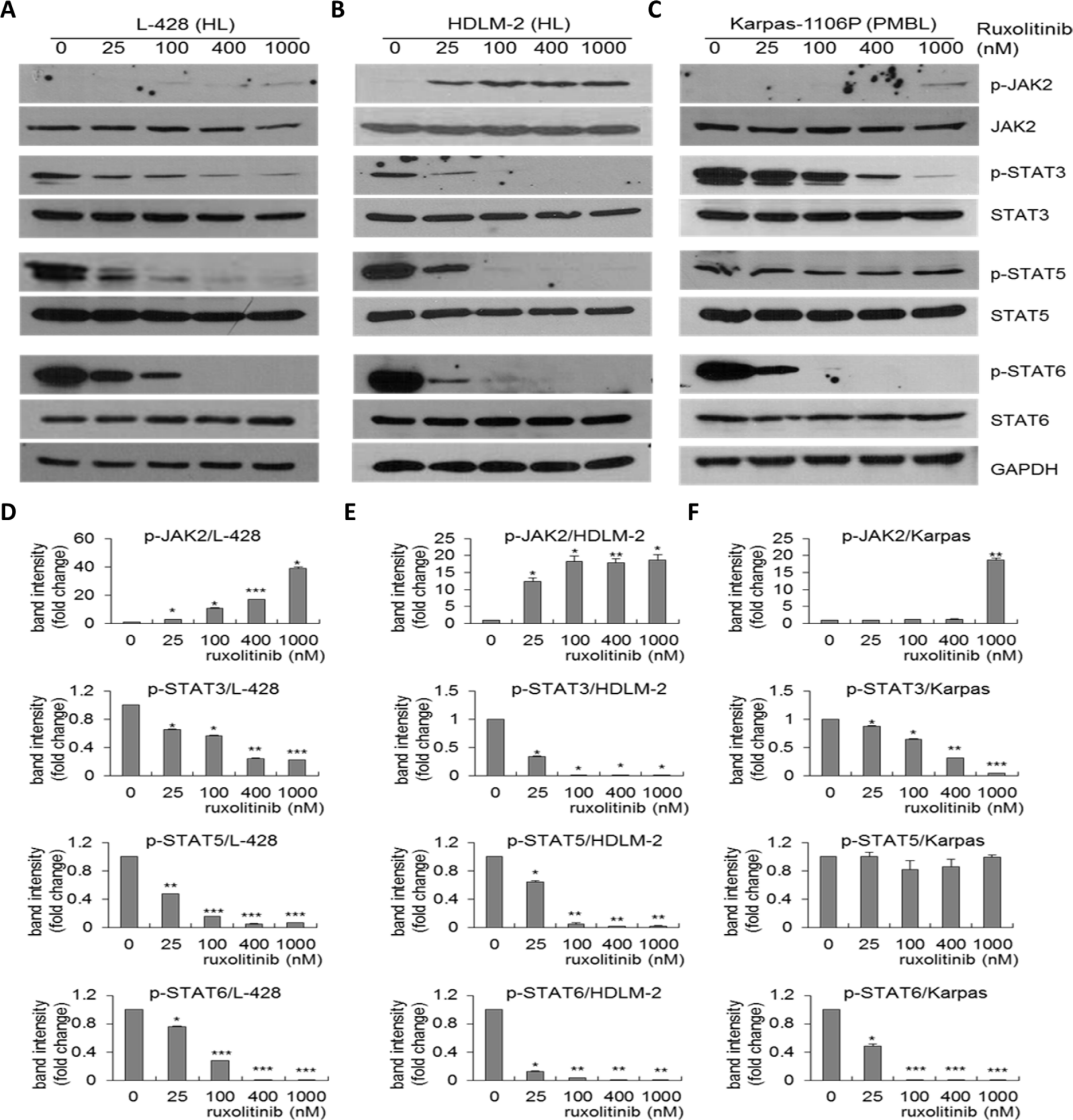
Downregulation of p-STAT3, p-STAT5 and p-STAT6 expression in HL and PMBL by ruxolitinib Total cell lysates were collected treated L-428 **(A)** and HDLM-2 **(B)** HL, and Karpas-1106P PMBL cells **(C)** following ruxolitinib treatment (0, 25, 100, 400, and 1000 nM), and western blotting of protein expression of p-JAK2, JAK2, p-STAT3, STAT3, p-STAT5, STAT5, p-STAT6 and STAT6 were performed. (*n =* 3). Band intensity was quantified by western blot in ruxolitinib treated L-428 **(D),** ^*^p < 0.0005;^**^p < 0.00005;^***^p < 0.00001) and HDLM-2 **(E)** HL, and Karpas-1106P PMBL cells **(F)**. Data are represented as the mean ± SD of triplicates (paired *t* test). ^*^p < 0.001;^**^p < 0.0001;^***^p < 0.00001.

To investigate the effects of ruxolitinib on the JAK2/STAT pathway in HL and PMBL cells, L-428 and HDLM-2 HL cells, and Karpas-1106P PMBL cells, each were treated with vehicle (dimethyl sulfoxide [DMSO]) alone or ruxolitinib (0, 25, 100, 400 and 1000 nM) for 48 hours and western blots were performed. Ruxolitinib significantly downregulated phosphorylation of STAT3, STAT5 and STAT6 in L-428 (Figure 2A and Figure 2D), HDLM-2 (Figure 2B and Figure 2E) following 25 nM ruxolitinib treatment compared to DMSO control whereas the expression level of total STAT3, STAT5 and STAT6 did not significantly change. Similarly, we observed significant inhibition of phosphorylation of STAT3 and STAT6 in 25 nM ruxolitinib treated Karpas-1106P cells (Figure 2C and Figure 2F). However, there was no significant inhibition of phosphorylation of STAT5 in Karpas-1106P following ruxolitinib treatment.

### Significant anti-proliferative *in vitro* effects of ruxolitinib in HL and PMBL cells

**Figure 3:**
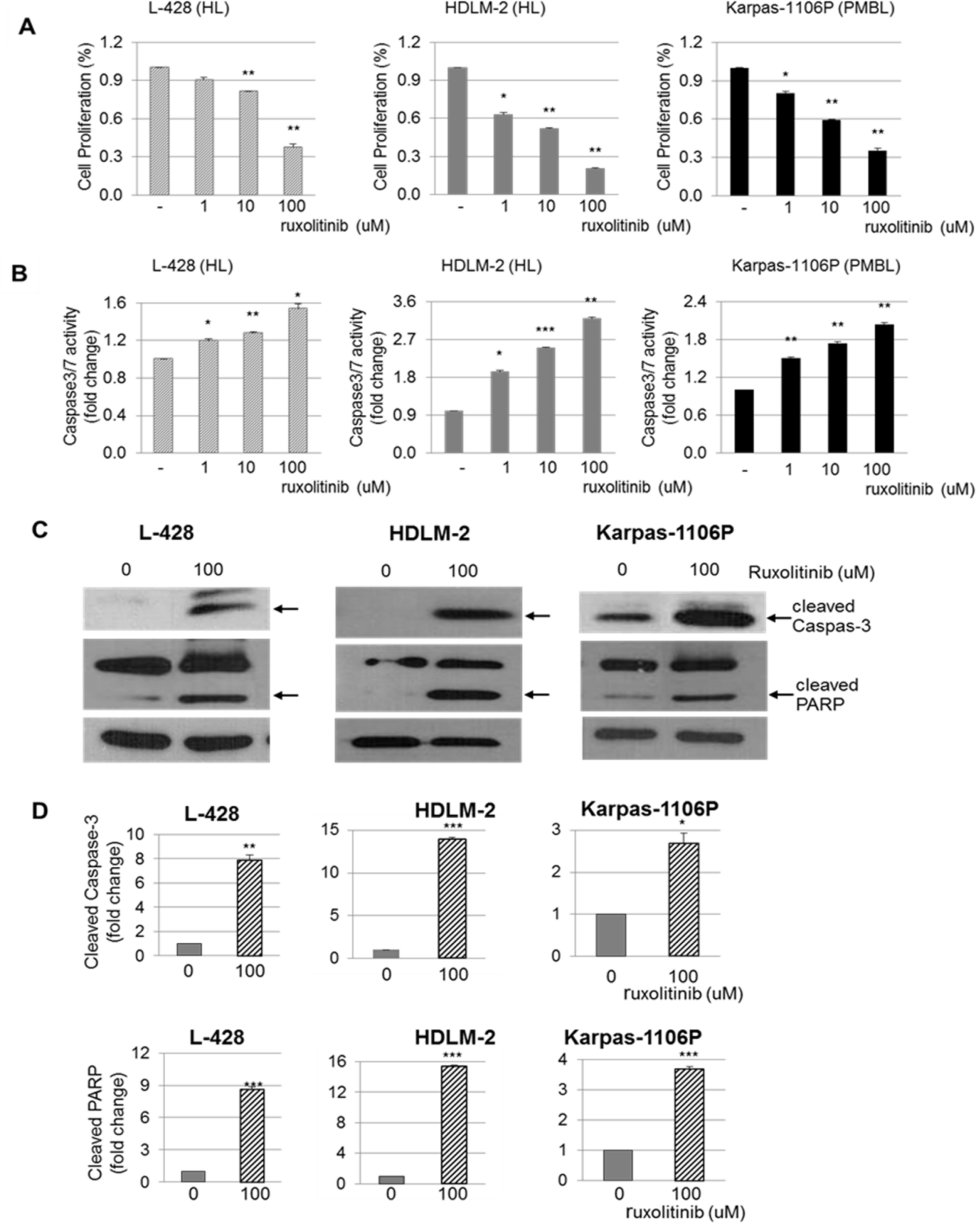
Cell proliferation, Caspase 3/7-dependent apoptosis and cleaved Caspas-3 and PARP in HL and PMBL cells Cells were plated (1x10^5^) into 48 well plates and cell growth was measured every 48 hours via MTS assay following ruxolitinib treatment (0, 1, 10 and 100 uM) in L-428 (left) and HDLM-2 (middle) HL cells, and Karpas-1106P PMBL cells (right) **(A)** (*n =* 3). Caspase 3/7 activity was measured using the Clarity Luminescence microplate reader (Biotek) in L-428 (left) and HDLM-2 (middle) HL cells, and Karpas-1106P PMBL cells (right) **(B)** (*n =* 3). Data are represented as the mean ± SD . ^*^p < 0.05; ^**^p < 0.01; ^***^p < 0.001. Cleaved Caspas-3 and PARP protein expression were measured by western blotting in L-428 (left) and HDLM-2 (middle) HL cells, and Karpas-1106P PMBL cells (right), and the arrow indicates cleaved Caspas-3 and PARP **(C)** (*n =* 3) and the intensity of the bands on gels by western blot was quantified in cells **(D)** (*n =* 3). Data are represented as the mean ± SD. ^*^p < 0.005; ^**^p < 0.001; ^***^p < 0.0005.

We examined the effect of ruxolitinib on HL and PMBL *in vitro* cell proliferation. Cells were treated with vehicle (DMSO) alone or ruxolitinib at various concentrations (0, 1, 10, and 100 uM) for 48 hours and cell proliferation was determined using MTS assay. There was a significant decrease in cell proliferation in the ruxolitinib treated L-428, HDLM-2 and Karpas-1106P *vs* control cells with DMSO treatment (20 - 79% reduction, p-value between *p <* 0.05 and *p <* 0.01) (Figure 3A). Specifically, there were significant decreases in cell proliferation with 20% reduction (*p <* 0.01) and 64% reduction (*p <* 0.01) with 10 and 100 uM of ruxolitinib treatment, respectively, in ruxolitinib treated L-428 cells. We also observed significant inhibition of cell proliferation with 38% (*p <* 0.05), 49% (*p <* 0.01) and 79% (*p <* 0.01) reduction in 1, 10 and 100 uM ruxolitinib treated HDLM-2 cells, respectively, compared to DMSO treated control cells. Ruxolitinib treated Karpas-1106P cells also showed significant inhibition with 20% (*p <* 0.05), 44% (*p <* 0.05) and 59% (0 < 0.05) reduction in 1, 10 and 100 uM ruxolitinib treated Karpas-1106P cells, respectively, compared to control cells following DMSO treatment.

### Significant increase in ruxolitinib-induced caspase-3/7 activity

To further investigate the mechanisms of the anti-proliferative activity of ruxolitinib, we determined the caspase-3/7 activity in ruxolitinib treated HL and PMBL cells. HL and PMBL cells were evaluated for fold change of caspase 3/7 activity over 48 hours with escalating doses of ruxolitinib (0, 1, 10 and 100 uM) (Figure 3B). There was a significant increase on caspase 3/7 activity at day 2 with >1.2 fold (*p <* 0.05), >1.3 fold (*p =* 0.01), and >1.5 fold (*p <* 0.05) increase at 1, 10, and 100 uM of ruxolitinib treatment, respectively, in L-428 cells compared to DMSO treated control cells. We also observed significant increases in caspase 3/7 activity in ruxolitinib treated HDLM-2 cells (>1.9 fold, *p <* 0.05; >2.5, *p <* 0.001; >3.2 fold, *p <* 0.01 in 1, 10 and 100 uM ruxolitinib, respectively). In addition, ruxolitinib significantly induced Caspase-3/7 activity in ruxolitinib treated Karpas-1106P cells (<1.5 fold, *p <* 0.01; >1.8 fold, *p <* 0.01; >2.0 fold, *p <* 0.01 in 1, 10 and 100 uM ruxolitinib, respectively) compared to control cells following DMSO treatment in Karpas-1106P cells. The cleavage of PARP (Figure 3C) and caspase-3 (Figure 3D), another hallmark of apoptosis, was significantly increased in ruxolitinib treated L-428 (*p <* 0.001 and *p <* 0.0005 at cleaved Caspase-3 and PARP, respectively), HDLM-2 (*p <* 0.0005 at both cleaved proteins) and Karpas-1106P cells (*p <* 0.005 and *p <* 0.0005 at cleaved caspase-3 and PARP, respectively) in the same dose-dependent manner following 100 uM of ruxolitinib treatment.

### Significant changes in the expression of pro- and anti-apoptotic protein and gene expression in ruxolitinib treated HL and PMBL cells

**Figure 4:**
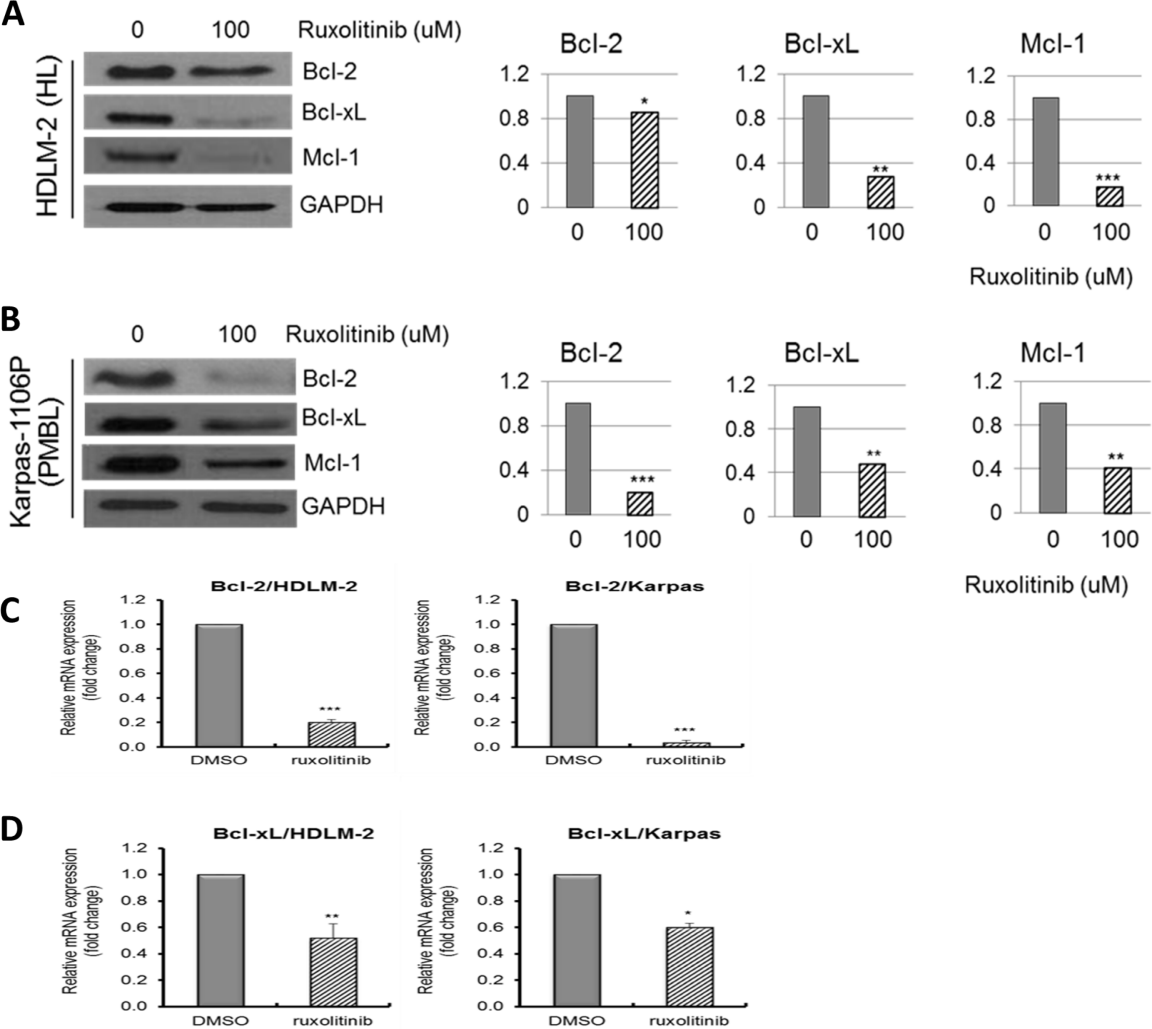
Downregulation of pro- and anti-apoptotic proteins and gene expression in HL and PMBL cells Ruxolitinib significantly induced apoptosis through down-regulation of anti-apoptotic proteins, Bcl-2, Bcl-xL and Mcl-1 in ruxolitinib HDLM-2 **(A)** (*n =* 3) HL and Karpas-1106P PMBL cells **(B)** (*n =* 3). The intensity of the bands on gels by western blot was quantified in cells. Data are represented as the mean ± SD. ^*^p < 0.005; ^**^p < 0.0005; ^***^p < 0.0001. Real time qRT-PCR expression levels of Bcl-2 mRNA **(C)** (*n =* 3) and Bcl-xL mRNA **(D)** (*n =* 3) in ruxolitinib treated HDLM-2 HL cells and Karpas-1106P PMBL cells are shown. GAPDH was used as endogenous control for qRT-PCR normalization. ^*^p < 0.01;^**^p < 0.005; ^***^p < 0.0005.

We also examined the effects of ruxolitinib on the expression of pro- and anti-apoptotic proteins to enhance our understanding of the effects of ruxolitinib on apoptosis in HL and PMBL cells. Cells were treated with 100 uM ruxolitinib for 48 hours and the expression of anti-apoptotic proteins such as Bcl-2, Bcl-xL and Mcl-1 were analyzed. There was significant decrease of Bcl-2 (*p <* 0.005), Bcl-xL (*p <* 0.0005) and Mcl-1 (*p <* 0.0001) in 100uM ruxolitinib treated HDLM-2 cells compared to DMSO treated cells (Figure 4A). Additionally, we observed significant inhibition of the protein expression of Bcl-2 (*p <* 0.0001), Bcl-xL and Mcl-1 (*p <* 0.0005) following 100 uM ruxolitinib exposure to Karpas-1106P cells (Figure 4B). We also observed significant inhibition of expression of Bcl-2 mRNA in 100 uM ruxolitinib treated HDLM-2 (*p <* 0.0005) and Karpas-1106P cells (*p <* 0.0005) (Figure 4C). Interestingly, ruxolitinib also significantly inhibited the expression of Bcl-xL mRNA in 100 uM ruxolitinib treated HDLM-2 (*p <* 0.005) and Karpas-1106P cells (*p <* 0.001) (Figure 4D). These results suggest that ruxolitinib decreases HL and PMBL survival by in part inducing programmed cell death via down-regulating the expression of pro- and anti-apoptotic genes in HL and PMBL cells.

### Effect of ruxolitinib on survival in PMBL and HL xenografted NSG mice

**Figure 5:**
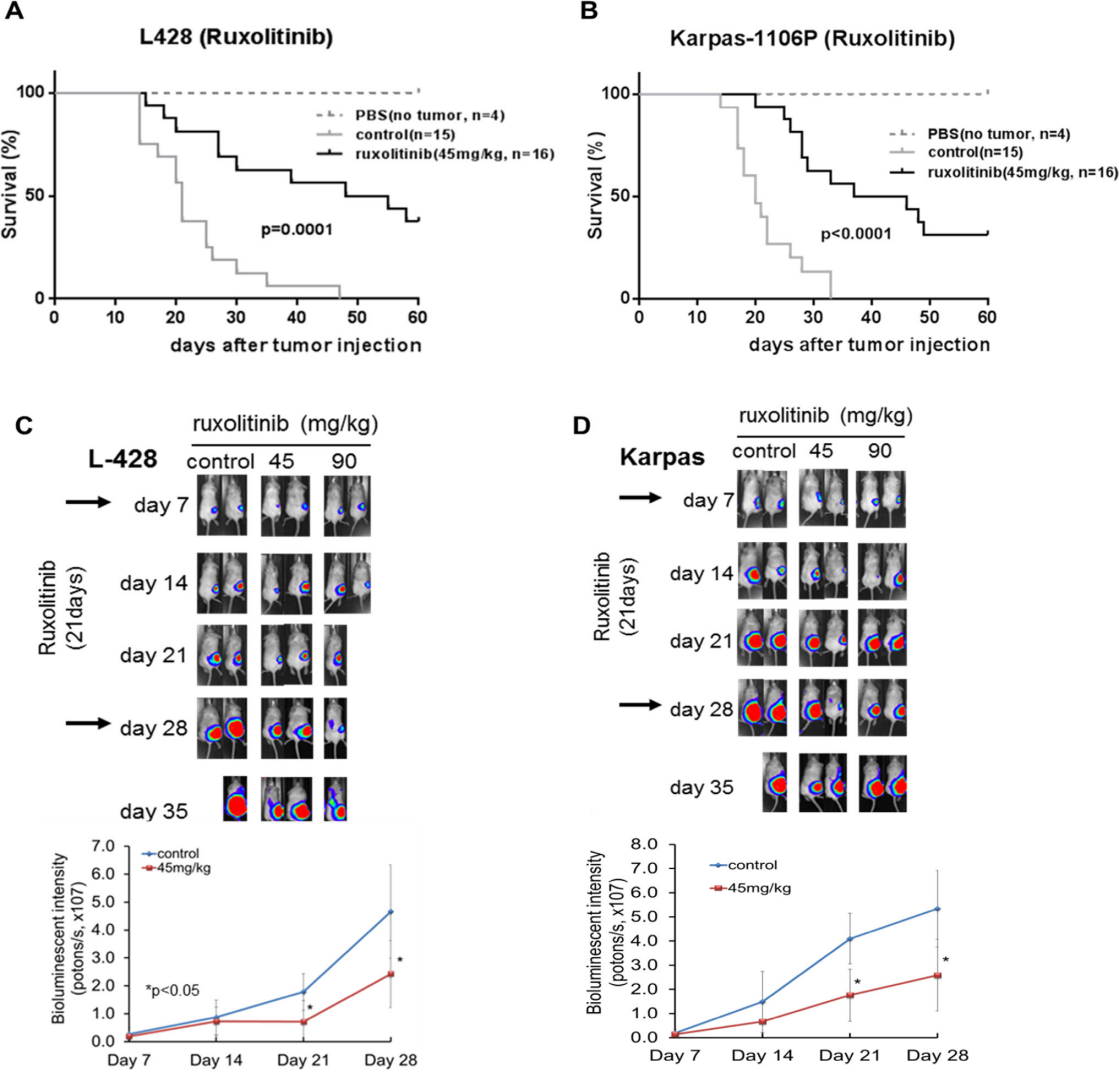
Survival in ruxolitinib treated HL and PMBL xenografted NSG mice Survival rates were analyzed following ruxolitinib treatment (45 mg/kg) by Kaplan-Meier method and differences evaluated by log-rank test. A significant difference in ruxolitinib treated L428 HL cells xenografted NSG mice (*n =* 16) compared to control (*n =* 15, *p =* 0.0001) **(A).** Ruxolitinib treated Karpas-1106P xenografted NSG mice (*n =* 16) had significantly extended survival time compared to the control mice (*n =* 15, *p <* 0.0001) **(B)**. A significant decrease in tumor luminescence intensity following ruxolitinib treated HL 28 (45 mg/kg, *p <* 0.05) **(C)** and PMBL (45 mg/kg) (*p <* 0.05) xenografted NSG mice **(D)** at day 21 and compared to control.^*^p < 0.05.

We further examined the efficacy of ruxolitinib in L-428 HL cells xenografted NSG mice and in Karpas-1106P PMBL cells xenografted NSG mice. Most importantly, ruxolitinib (45.0 mg/kg) treated L-428 (*p <* 0.03) and Karpas-1106P (*p <* 0.002) xenograft NSG mice had a significantly prolonged survival compared to control mice. Finally, we observed that ruxolitinib (45.0 mg/kg) treated L-428 xenografted NSG mice (*n =* 16) had significantly prolonged survival time with a median of 51.5 days compared to control mice (*n =* 15) (21 days, *p =* 0.0001) (Figure 5A), along with significant prolonged survival in ruxolitinib treated Karpas-1106P xenografted NSG mice (*n =* 16, median of 41.5 days) compared to control mice (*n =* 15, median of 20 days) (*p <* 0.0001) (Figure 5B). Furthermore, we demonstrated a significant decrease in L-428 HL and Karpas-1106 PMBL in bio-luminescence intensity (tumor burden) following ruxolitinib treatment in HL xenografted NSG mice (45.0 mg/kg, *p <* 0.05) at day 21 and 28 (Figure 5C), and Karpas-1106P PMBL cells xenografted NSG mice (45.0 mg/kg) (*p <* 0.05) at day 21 and 28 compared to control mice (Figure 5D).

## DISCUSSION

We investigated the efficacy of ruxolitinib in HL and PMBL cells and found significant *in vitro* anti-proliferative and proapoptotic effects of ruxolitinib treated L-428 and HDLM-2 HL cells and Karpas-1106P PMBL cells. Additionally, we observed significant downregulation in the phosphorylation of STAT3, STAT5 and STAT6 in HL, and p-STAT3 and STAT6 in PMBL following ruxolitinib treatment. Most importantly, ruxolitinib significantly decreased tumor burden and prolonged survival in HL and PMBL xenografted NSG mice.

HL and PMBL are highly curable diseases in first complete remission; however, patients with relapsed or refractory HL and PMBL have typically been associated with high failure rates despite aggressive chemo-radiotherapeutic approaches and/or autologous stem cell marrow transplantation [6, 33–35]. Furthermore, late effects in both HL and PMBL are significant, which affect both morbidity and mortality. Thus, novel salvage regimens are needed and less toxic new approaches in newly diagnosed patients are also warranted. Patients with the myelofibrosis (MF) carrying activating mutant allele of JAK2 exhibited rapid, significant reduction in disease burden and durable clinical benefit in patients following ruxolitinib [31, 36].

Ruxolitinib is an orally available pyrrolo [2,3-d] pyrimidine analog that exhibits subnanomolar affinity for JAK2 and JAK1 but exhibits lower activity against JAK3 [30]. Our observations in this study are consistent with these findings that ruxolitinib has selective activity of JAK2 against JAK3 protein, as demonstrated by inhibition of phosphorylation of the downstream STAT3 and STAT5 proteins at 10-100 nM ruxolitinib in HDLM-2 JAK2/STAT signaling *vs* L-540 JAK3/STAT signaling. In agreement with earlier studies, increased levels of JAK2 phosphorylation were observed despite inhibition of downstream STA3, STAT5 and STAT6 signaling in HL and PMBL cells. Similar paradoxical phosphorylation has been previously reported after incubation with JAK2 inhibitor Gö6976 and SB1518 in primary acute myeloid leukemia cells HEL92.1.7, [37, 38] and JAK inhibitor AZD 1480 in HL cells [39]. These findings could be related to the induction of negative-feed-back loops involving activating cytokines. Suppressor of cytokine signaling 1(*SOCS1*) works as the key effector of a classic negative feedback loop in JAK/STAT signaling. SOCS1 has been identified as a recurrently mutated tumor suppressor gene in HL and Karpas-1106P PMBL cells [40, 41]. Further, mutated SOCS1 may have contributed to the hyper-phosphorylation of JAK2 in HL and PMBL. Finally, ruxolitinib has also been demonstrated to inhibit the phosphorylation of ERK1/2, especially downstream to RAS-RAF-MEK-ERF signaling pathway [30, 42].

In this study, we demonstrated that ruxolitinib significantly inhibits HL and PMBL JAK/STAT signaling pathways. Furthermore, ruxolitinib significantly suppresses cell proliferation in HL and PMBL cells. This is consistent with inhibition of cell proliferation reported by Ju *et al.* in 4 HL cell lines [43]. The half maximal inhibitory concentration (IC_50_) is shown for L-428 (IC_50_ = 74.9 uM), HDLM-2 (IC_50_ = 15.7 uM) and Karpas-1106P (IC_50_ = 43.8 uM). Compared to the abrogation of downstream STAT phosphorylation by ruxolitinib at sub-micromolar and micromolar concentrations, relatively less sensitive anti-proliferative effects were observed. The relatively high IC_50_ of HL and PMBL cells imply that SOCS1 negative-feedback loop may activate secondary signaling pathways other than JAK2/STAT in HL and PMBL.

The maximum tolerated dose (MTD) for ruxolitinib in MF was established at 25 mg twice daily or 100 mg once daily [31, 44]. When investigated in children with relapsed/refractory malignancies in a phase 1 consortium study by the Children’s Oncology Group at five dose levels, ruxolitinib did not exceed the MTD; dose limiting toxicity (DLT) was observed in ≤1 of 6 patients per cohort [45]. In a phase I/II study of ruxolitinib in patients >14 years old with relapsed/refractory acute myeloid leukemia (AML), ruxolitinb was reasonably well tolerated in doses ranging from 50 mg twice daily to 200 mg twice daily [46]. In the present study, while we saw some effects at the lower concentration, the effect was augmented with higher dosing. These findings, with the relatively high IC_50_ in HL and PMBL cells in our study, suggest that higher dosing of ruxolitinib may be necessary for optimum activity, and may be well tolerated.

A kinetic analysis of caspase 3/7 activity following ruxolitinib therapy in HL and PMBL cells revealed that ruxolitinib significantly activates caspase 3/7 and induces apoptosis. This is in part presumably secondly to down- regulating the expression of anti-apoptotic factors, such as Bcl-2 and Bcl-xL in HL and PMBL cells. Our results suggest that therapy with ruxolitinib significantly decreased tumor burden and prolonged survival in HL and PMBL xenografted NSG mice. This is consistent with *in vitro* and *in vivo* JAK2 inhibition in HL and MLBL using fedratinib [26, 29]. Kim *et al.* demonstrated in a phase I study the safety and efficacy of ruxolitinib in adult patients with HL and PMBL, with disease control achieved in 54% (7/13) patients with heavily pretreated relapsed or refractory HL [47]. Most recently, Neste *et al.* demonstrated in a phase II study, a best ORR (1 CR/5 PR) of 19% (6/32) of ruxolitinib in adults with relapsed/refractory HL [48]. These results are consistent with our preclinical studies of mild to modest activity.

In summary, we have demonstrated that ruxolitinib, a JAK1/JAK2 inhibitor, significantly blocks *in vitro* STATs signaling, induces apoptosis and inhibits cell proliferation *in vitro* against HL and PMBL cell lines and *in vivo* prolonged survival in HL and PMBL xenografted NSG mice. Thus ruxolitinib has the potential to be an alternative therapeutic strategy in patients with high risk HL and PMBL. In PMBL, in addition to gains of chromosome 9p (including *JAK2, JMJD2C, PDL1, PDL2*), gain of chromosome 2p (including *REL, BCL11A*) has been consistently reported in up to 50% of patients [49, 50]. Gains of the same regions are also detected in HL [19, 51]. These genetic features suggest that there may be options to improve the therapeutic effect of ruxolitinib by combining it with other targeted therapies such as PD-1 or PDL-1 blockades or brentuximab-vedotin, in cHL [26, 43].

Future studies will be required to determine how best to incorporate this therapeutic agent in both newly diagnosed high risk and in relapsed/refractory patients with both HL and PMBL.

## MATERIALS AND METHODS

### Cell lines

The HL cell lines HDLM-2, L-428, L-540 and PMBL cell line Karpas-1106P cells were obtained from the German Collection of Microorganisms and Cell Cultures (DSMZ, Germany). All of these 4 cell lines were previously demonstrated to have a significant 9p24.1 and JAK2 amplification [25]. The L-428 cells were cultured in RPMI 1640 medium supplemented with 10% heat-inactivated fetal bovine serum (GIBCO BRL, Gaithersburg, MD, USA) 1% glutamine and penicillin-streptomycin in a humid environment of 5% CO_2_ at 37° C. The HDLM-2, L-540 and Karpas-1106P cell lines were cultured in RPMI 1640 medium supplemented with 20% heat-inactivated fetal bovine serum.

### Cell proliferation assay

Cell growth was determined by the Cell titer 96 Aqueous One solution cell proliferation assay (MTS) (Promega, Madison, WI). At 24, 48 and 72 hours after treatment with INC424 or DMSO, MTS reagent was added and cells were incubated for 1 hour at 37° C. Cell proliferation was measured by OD 490 nm using Multilabel Counter (Perkin Elmer, Massachusetts, USA).

### Caspase 3/7 assay

Caspase 3/7 activity was directly measured at 24, 48 and 72 hours after treatment using Caspase-Glo 3/7 Activity kit (Promega) as per the manufacturer’s protocol. Briefly, the cells (5 × 10^5^/ml) were seeded into 24-well plated and treated with vehicle (DMSO) alone or ruxolitinib at various concentrations for 48 and 72 hours, Caspase-Glo reagent was added and cells were incubated for 1 hour at room temperature in the dark. Relative light intensity was measured in each well using Clarity Luminescence microplate reader (BioTek, Vermont, USA).

### Reagents and antibodies

Ruxolitinib (INC424) was generously provided by Incyte Corporation (Wilmington, DE, USA. AG-490). Antibodies specific for phospho-JAK3, JAK3, STAT3, STAT5, were purchased from Santa Cruz Biotechnology (Santa Cruz, CA, USA). Antibodies specific for phospho-STAT3, phospho-STAT5, phospho-STAT6, STAT6, phospho-JAK2, JAK2, Bcl-xL, Bcl-2, Mcl-1, Caspas-3, poly adenosine diphosphate ribose polymerase (PARP) were purchased from Cell Signaling Technology (Cambridge, MA, USA).

### Western blot analysis

Cell pellets were suspended in a lysis buffer containing 50 mmol/l Tris–HCl, pH 7.4, 350 mmol/l NaCl, 1% TritonX-100, 0.5% Nonidet P-40, 10% glycerol, 0.1% sodium dodecyl sulfate (SDS), 1 mmol/l EDTA, 1 mmol/l EGTA, 1 mmol/l Na3VO4, 1 mmol/l phenylmethylsulphonyl fluoride (PMSF) and phosphatase inhibitor cocktails on ice. Whole-cell extracts were resolved on SDS-PAGE, transferred to nitrocellulose membrane, and probed with appropriate antibodies. Membranes were blocked in 5% non-fat dried milk in Tris-buffered saline (pH 7.4) containing 0.1% Tween 20 (TBST) for 1 hour and subsequently incubated with primary antibodies diluted in TBST at 4° C overnight. Membranes were then probed with horseradish Peroxidase-conjugated secondary antibodies and developed using enhanced chemiluminescence (ECL) reagent (GE Healthcare Bio-Sciences, Piscataway, NJ, USA). Band intensities on SDS-PAGE gel were measured using ImageJ software program [52].

### Quantitative reverse-transcriptase polymerase chain reaction (qRT-PCR)

Total RNA was prepared using Trizol reagent (Invitrogen) according to the manufacturer’s directions, and cDNA was synthesized using qScript™ cDNA Synthesis Kit (Quantas) using 1ug of RNA. The qRT-PCR was performed using CFX96 Real-time system (Bio-rad) and SsoFast™ EvaGreen^®^ Supermix (Bio-rad) as PCR reagents. Relative quantification (ddCt) of mRNA expression of genes was determined by normalizing to the housekeeping gene (GAPDH). Primers used in qRT-PCR are previously described [53].

### *In vivo* xenograft mouse models

The experimental animal protocols, procedures and care were approved by the Institutional Animal Care and Use Committee at New York Medical College (NYMC), Valhalla, NY, USA. Kapas-1106P PMBL cell line and L-428 HL cell line were stably transfected with a firefly luciferase expression plasmid (*ffluc-zeo*), kindly provided by Laurence Cooper MD, PhD, and then stable clones were selected under zeocin (Invitrogen) selection. The mice were **γ**-irradiated (2.5 Gy) 1 day before tumor cell transplantation. Six- to eight- week-old female NSG (NOD.Cg-Prkdcscid *Il2rg*^*tm1Wjl*^/SzJ) mice (The Jackson laboratory, Bar Harbor, ME) were subcutaneously injected with 1 × 10^6^ ffluc-zeo Karpas-1106P or L-428 cells. The tumor burden was verified by bioluminescence imaging (BLI) system using the Xenogen IVIS-200 (Caliper Life Sciences) for up to 50 days as we have previously described [54]. Mice were orally gavaged with either 0.5% methyl cellulose vehicle or ruxolitinib (45.0 mg/kg) for 21 days. Tumor progression was monitored at day 7 and once every week by BLI. The survival rate was analyzed using humane endpoints and all mice were sacrificed when tumor size was greater than 2.0 cm^3^ ([length × width2]/2]) or with signs of ulceration, or if mice were moribund.

### Statistical analysis

Data obtained from independent experiments are represented as means ± standard deviation (SD) and significant differences between two groups were determined by using Student’s *t-test*. Statistical analysis was performed using a two-tailed Student’s *t* test. P values less than 0.05 were considered significant. *In vivo* study, survival rates were analyzed by the Kaplan-Meier method and differences evaluated by log-rank test using the Prism Version 6.0 software.

## Abbreviations

AML: acute myeloid leukemia
AYA: adolescents and young adults
BID: bis in die (twice daily)
BLI: bioluminescence imaging
DLBCL: diffuse large B-cell lymphoma
DLT: dose limiting toxicity
DMSO: dimethyl sulfoxide
ECL: enhanced chemiluminescence
EFS: event-free survival
Ffluc: firefly luciferase
HL: Hodgkin lymphoma
JAK: Janus Kinase
MF: myelofibrosis
MLBL: mediastinal large B-cell lymphoma
MPNs: myeloproliferative neoplasms
MTD: maximum tolerated dose
NFKB: nuclear factor kappa B
NSG: non-obese diabetic severe combined immunodeficiency gamma
NYMC: New York Medical College
PDL-1: programmed death ligand-1
PMBL: primary mediastinal large B-cell lymphoma
qRT-PCR: Quantitative reverse-transcriptase polymerase chain reaction
SD: Standard deviation
SOCS1: Suppressor of cytokine signaling 1
STAT: signal transducer and activator of transcription
TBST: Tris-buffered saline Tween
